# 

**Published:** 1918-07-06

**Authors:** 


### Manager's Notices.

**All letters on business matters must be addressed to The Manager, "The Hospital," 28 and 29 Southampton Street, London, W.C. 2.**

**Special Rates** will be quoted to those institutions that agree to send all their public notices to The Hospital, so as to make it a file of such announcements for ready reference.

Rates of Subscription (payable in advance).

| | |
|---|---|
| UNITED KINGDOM. | |
| Three Months (including Postage) | 2s. 9d. |
| Six Months do. | 5s. 5d. |
| Twelve Months do. | 7s. 9d. |
| FOREIGN AND COLONIAL. | |
| Three Months (including Postage) | 3s. 3d. |
| Six Months do. | 5s. 6d. |
| Twelve Months do. | 13s. 0d. |

To obtain **The HOSPITAL**
regularly **EVERY THURSDAY** a definite order **must** be placed with your Newsagent **at once.** All Newsagents, Booksellers, and Railway Bookstalls will gladly book your order, but copies cannot be stocked for sale to chance customers.

### Matrons' and Sisters' Appointments.

Red Cross Hospital, Perth.—Sister Kerrigan has been appointed night sister. She was trained at the Royal Infirmary, Edinburgh, and has been theatre sister at Townley's Military Hospital, Bolton, and assistant matron at the District Asylum, Larbert.

Royal Surrey County Hospital, Guildford.—Miss R. A. Longland has been appointed matron. She was trained at the Great Northern Central Hospital, where she was later sister and night superintendent. She has also been sister at the Norfolk War Hospital, and assistant matron and acting matron at the Royal Surrey County Hospital.

Royal Victoria and West Hants Hospital, Boscombe.—Miss Vera Hollick has been appointed ward sister. She was trained at the Cheltenham General Hospital and at the City of London Lying-in Hospital; has been ward sister at the Alexandra Hospital for Children with Hip Disease, Queen Square, and at Salisbury Infirmary, and has been nursing under the Victorian Order in Canada.

Welsh Hospital, Netley.—Miss Kathleen S. Stewart, A.R.R.C., has been appointed matron. She was trained at the Royal Infirmary, Sunderland, and at the Royal Maternity Hospital, Edinburgh. She has since been district and ward sister at the Deaconess Hospital, Edinburgh; night superintendent and housekeeping sister at the Royal Infirmary, Sunderland; housekeeping sister at Charing Cross Hospital; assistant matron at the Royal Hospital for Sick Children, Edinburgh; and matron of the York County Hospital.

Woodlands Auxiliary Hospital, Rawdon, Leeds.—Miss J. H. Stableforth has been appointed sister. She was trained at the East Suffolk and Ipswich Hospital, and has been sister at the General Hospital, Chelmsford, and at the Kent and Canterbury Hospital.

**Note.—Particulars of Appointments for insertion in this column will be welcomed.**

### The Book Market.

LIST OF NEW BOOKS RECEIVED FROM THE FOLLOWING PUBLISHERS:—

Jarrolds, Ltd.: "Ordeal by Sea." By Archibald Hurd. 5s. net.

John Murray: "With the Scottish Nurses in Rumania." By Yvonne Fitzroy. 5s. net. "My War Diary." By Mary King Waddington. 6s. net.

Butterworth and Co. (India), Ltd.: "Painless Childbirth in Twilight Sleep in the East." By Cecil Webb-Johnson. Rs. 4 net.

Duckworth and Co.: "Medical Contributions to the Study of Evolution." By J. G. Adami, M.D. 18s. net.

Wm. Heinemann: "The New Book of Martyrs." By Georges Duhamel. 3s. 6d. net. "The War-Workers." By E. M. Delafield. 6s. net.

Simpkin, Marshall, Hamilton, Kent and Co., Ltd.: "The Happy Hospital." By Cpl. Ward Muir, R.A.M.C. (T.). 1s. 6d. net. "Many Thanks—Ben Hamett." By Herbert de Hamel. 1s. 6d. net.

J. M. Dent and Sons, Ltd.: "Dent's Medical Dictionary." By W. B. Drummond, M.B., C.M., F.R.C.P. (Edin.). 10s. 6d. net.

The Scientific Press, Ltd: "First Aid for Foot Troubles, with Special Reference to Battalion Chiropody." By Ernest G. V. Runting. 1s. 3d. net.

Incorporated Sanitary Association of Scotland: "Transactions of the Incorporated Sanitary Association of Scotland, 1917."

### Recent Official Publications.

(To be obtained from H.M. Stationery Office, Imperial House, Kingsway, W.C. 2.)

The Inter-Allied Conference on the After-Care of Disabled Men. Second Annual Meeting held in London. Post free 5s. 5d.

National Health Insurance. Inquiries as to Medical Practitioners' and Chemists' Regulations, 1918. Draft Regulations. Post free 1½d. Women's Equalisation Fund Regulations, 1918. Draft Regulations, June 18, 1918. Post free 1½d. National Insurance Act, 1911 (Part I.), Account. H.C.65/1918. Post free 1½d. Inquiries as to Medical Practitioners and Chemists' Regulations (Wales), 1918. Draft Regulations, May 16, 1918. Post free 1½d. Appeal from Insurance Committee Audit Regulations (Wales), 1918. Draft Regulations, May 16, 1918. Post free 1½d. Collection of Contributions Consolidated Regulations (Wales), 1918. Draft Regulations, May 16, 1918. Post free 4d. Insurance Committees (Amendment) Regulations (Ireland), 1918. Draft Regulations, June 7, 1918, further extending the Term of Office of Members of Insurance Committees. Post free 1½d.

Injuries and Diseases of War. A Manual Based on Experience of the Present Campaign in France. January, 1918. Post free 1s.

List of Sanatoria and other Residential Institutions approved by the Local Government Board. Post free 2d.

Medical Supplement (compiled by the Medical Research Committee) to the Daily Review of the Daily Press. Vol. I., No. 6. Post free 1s. 3d.

Fourth Report of the Highlands and Islands Medical Service Board for 1917. Post free 3d.

Midwives Bill. H.L.39/1918. Post free 3d. Amendments to be moved in Committee. H.L.39A/1918. Post free 1½d. Amendment. H.L./39B/1918. Post free 1½d.

Reports of the Local Government Board on Public Health and Medical Subjects. Statistics of the Incidence of Notifiable Infectious Diseases in each Sanitary District. Post free 1s.

Fourth Annual Report of the General Board of Control for Scotland. Cd.9068. Post free 4d.

Ministry of Munitions. Health of Munition Workers Committee. Final Report. Industrial Health and Efficiency. Cd.9065. Post free 2s. 4d.

Local Government Board. Dried Milk for Infants. Résumé of a Report to the Local Government Board. Post free 1½d.

### Lectures on Venereal Disease.

A Beginning at Manchester.

A short course of lectures has been arranged by the Manchester branch of the National Council for Combating Venereal Diseases. The lectures, which are mainly intended for welfare and social workers, and intending speakers on the subject, are being conducted at the Medical School, the University, and the first of the series was delivered on May 28 by Dr. Dean, Professor of Pathology at the University. He dealt with the main symptoms of the diseases and the essential importance of obtaining skilled treatment at the outset. Microscopic slides were shown of blood tests containing gonococci and the *Spirochetæ pallida.*

